# Case management after acquired brain injury compared to care as usual: study protocol for a 2-year pragmatic randomized controlled superiority trial with two parallel groups

**DOI:** 10.1186/s13063-020-04804-2

**Published:** 2020-11-17

**Authors:** Annemarie P. M. Stiekema, Christine Resch, Mireille Donkervoort, Natska Jansen, Kitty H. M. Jurrius, Judith M. Zadoks, Caroline M. van Heugten

**Affiliations:** 1 Department of Psychiatry and Neuropsychology, School of Mental Health and Neuroscience, Maastricht University, Maastricht, The Netherlands; 2 Limburg Brain Injury Center, Maastricht University, Maastricht, The Netherlands; 3 Department of Neuropsychology and Psychopharmacology, Faculty of Psychology and Neuroscience, Maastricht University, Maastricht, the Netherlands; 4 Health Care and Social Work Division, Windesheim University of Applied Sciences, Almere, The Netherlands; 5 Mevrouw Slimmer Werken Social Innovation in Health Care and Well-Being, Drogteropslagen, Netherlands; 6 Brain Injury Team, Overijssel, Netherlands; 7 In-Tussen Foundation, Utrecht, the Netherlands; 8 BreinDok Innovation in Care, Utrecht, the Netherlands

**Keywords:** Brain injury, Stroke, Traumatic brain injury, Caregivers, Case management, Transitional care, Psychosocial, Early intervention, Family, Randomized controlled trial

## Abstract

**Background:**

People with acquired brain injury may suffer from cognitive, emotional and behavioural changes in the long term. Continuity of care is often lacking, leading to a variety of unmet needs and hindering psychosocial functioning from the occurrence of brain injury up to years thereafter. Case management aims to prevent (escalation of) problems and to facilitate timely access to appropriate services. In other populations, case management has shown to improve psychosocial well-being. In this study, we aim to evaluate the feasibility of case management after acquired brain injury and its effectiveness and cost-effectiveness, compared to care as usual.

**Methods:**

This is a pragmatic randomized controlled superiority trial with two parallel groups and repeated measures in adults with ABI and their family, taking place between November 2019 and December 2021 in three provinces in the Netherlands. Participants will be randomly allocated to either the case management group, receiving case management from hospital discharge up to 2 years thereafter, or the control group, receiving care as usual. Effectiveness will be evaluated every 6 months for 18–24 months by patient-reported psychosocial well-being (Hospital Anxiety and Depression Scale (HADS), Utrecht Scale for Evaluation of Rehabilitation-Participation (USER-P) restriction subscale and the Life Satisfaction Questionnaire (LiSat)), self-management (Patient Activation Measure (PAM)) and care needs (Longer-term Unmet Needs after Stroke (LUNS)). Family outcomes include self-efficacy (Carer Self-Efficacy Scale (CSES)), caregiver burden (Caregiver Strain Index (CSI)), psychosocial well-being (LiSat, HADS), family needs (Family Needs Questionnaire (FNQ)). Feasibility will be evaluated using qualitative methods, assessing fidelity, dose delivered, dose received, reach, recruitment and context. Cost-effectiveness will be determined by the EQ-5D-3L and service use.

**Discussion:**

At the moment, there is no integrated health care service for people with acquired brain injury and their family members in the long term. If case management is shown to be feasible and (cost)-effective, it could bridge the gap between patients’ and families’ needs and the available services.

**Trial registration:**

Netherlands Trial Register NL8104. Registered on 22 October 2019.

## Administrative information

The order of the items has been modified to group similar items (see http://www.equator-network.org/reporting-guidelines/spirit-2013-statement-defining-standard-protocol-items-for-clinical-trials/).

**Table:** 

Title {1}	Case management after acquired brain injury compared to case as usual: study protocol for a two-year pragmatic randomized controlled trial
Trial registration {2a and 2b}.	Netherlands Trial Register, registration number NL8104
Protocol version {3}	Version 3, 17 September 2019
Funding {4}	Ministry of Health, Welfare and Sport (Dutch: Ministerie van Volksgezondheid, Welzijn en Sport).
Author details {5a}	**Annemarie P.M. Stiekema**, Department of Psychiatry and Neuropsychology, School of Mental Health and Neuroscience, Maastricht University, Maastricht, The Netherlands; Limburg Brain Injury Center, Maastricht University, Maastricht, The Netherlands **Christine Resch**, Department of Neuropsychology and Psychopharmacology, Faculty of Psychology and Neuroscience, Maastricht University, Maastricht, the Netherlands; Limburg Brain Injury Center, Maastricht University, Maastricht, The Netherlands **Mireille Donkervoort** Health Care and Social Work Division, Windesheim University of Applied Sciences, Almere, The Netherlands. **Natska Jansen** Mevrouw Slimmer Werken social innovation in health care and well-being, Drogteropslagen, Netherlands; Brain injury team Overijssel, Netherlands. **Kitty H.M. Jurrius** Health Care and Social Work Division, Windesheim University of Applied Sciences, Almere, The Netherlands. **Judith M. Zadoks** In-Tussen Foundation, Utrecht, the Netherlands; BreinDok Innovation in Care, Utrecht, the Netherlands. Caroline M. van Heugten*, Department of Psychiatry and Neuropsychology, School of Mental Health and Neuroscience, Maastricht University, Maastricht, The Netherlands; Limburg Brain Injury Center, Maastricht University, Maastricht, The Netherlands; Department of Neuropsychology and Psychopharmacology, Faculty of Psychology and Neuroscience, Maastricht University, Maastricht, The Netherlands; Limburg Brain Injury Center, Maastricht University, Maastricht, The Netherlands *Corresponding author
Name and contact information for the trial sponsor {5b}	Maastricht University, Maastricht, the Netherlands
Role of sponsor {5c}	The sponsor and funder had no role in the study design and will have no role in the collection, management, analysis, and interpretation of data nor in the decision to submit the report for publication.

## Introduction

### Background and rationale {6a}

Acquired brain injury (ABI) may result from stroke (e.g. ischemic or haemorrhagic disruption of blood flow), traumatic brain injury (e.g. from a fall or a blow to the head), brain disease or hypoxia (e.g. after cardiac arrest or near-drowning). People with ABI often experience physical, communicative, cognitive, emotional or behavioural problems [1–3]. The persisting nature of these changes poses day-to-day challenges in a variety of life domains, such as work or education, household, social relationships and leisure [4–6], affecting not only quality of life of people with ABI but that of family members as well, as they may need to take on the role of caregiver [3, 7, 8]. There are ample health care services available for people with ABI, but problems exist with regard to their continuity, accessibility and timing [9–11]. People with ABI and family members feel ill-prepared for discharge from the hospital or rehabilitation centre and ‘abandoned’ once at home, being left with unmet health, social and vocational needs in the long term [10, 11].

The importance of supporting a changed life after ABI is increasingly recognized in clinical guidelines [12, 13]. Since there are relatively few methodologically sound studies evaluating longer-term care, the Action Plan for Stroke in Europe and the World Stroke Organization state that the development and evaluation of a ‘seamless, coordinated chain of support’, which includes life after ABI, is a development and research priority [14, 15].

The development and research on longer-term care services for ABI falls behind in comparison to populations where the need for long-term support has been recognized for longer, such as dementia, oncology and diabetes. A form of longer-term support for these populations is case management, which focusses on supporting people to adapt to the consequences of their health condition in daily life [16–20]. Case management promotes self-management, which refers to choosing strategies, making decisions and undertaking activities to manage a long-term condition and its consequences [21]. Case management varies in form and duration. The key element is a professional, the case manager, who serves as a first point of contact for patients and their family, is familiar with their situation, supports independent living and links them to available services in the community [22]. Case management has a positive impact on well-being in dementia, oncology and diabetes, reducing anxiety and depression and increasing quality of life [16–20]. It may decrease financial strains on healthcare as well; for dementia, costs were reduced by 22–33% when providing case management compared to care as usual [23].

Case management for ABI has been described in the literature, and commonly involves engagement, assessment, planning, education, training and skills development, emotional and motivational support, advising, coordination and monitoring [24]. These elements are based on best practice; to the best of our knowledge, no randomized controlled trials on long-term case management for ABI have been undertaken to date. The evidence base so far is weak, with a few relatively old non-randomized studies on case management for traumatic brain injury of too low a quality to draw conclusions on its effectiveness [25–29]. Long-term follow-up did show a positive effect on social activities and depression in stroke in a non-randomized trial [30] and short-term transitional care interventions also show promising results [31, 32]. However, since learning how to live with ABI is a dynamic process with fluctuating needs over the course of several years, 3 to 6-month follow-up in the first year and annual reviews hereafter are necessary [14]. A methodologically sound investigation of the feasibility and effects in terms of health and costs of such long-term support is called for [14, 15]. This article describes the study protocol for a pragmatic randomized controlled trial on long-term case management (18–24 months) for people with ABI and their family.

### Objectives {7}

The primary objective is to examine the effectiveness of case management for ABI compared to the care as usual on psychosocial well-being (emotional, participation and quality of life outcomes), self-efficacy and unmet needs. Secondary objectives are to explore cost-effectiveness and cost-utility (the balance of costs and gains in health and well-being) of case management compared to care as usual and to explore feasibility of case management for people with ABI and their family in terms of fidelity, dose delivered, dose received, reach and recruitment within its physical, social and political context. We expect case management to be effective and feasible, and we hypothesize that healthcare costs will rise at first and will be reduced in the long-term.

### Trial design {8}

This is a pragmatic prospective randomized controlled superiority trial with two parallel groups and repeated measures. Randomization will be performed as block randomization with a 1:1 allocation.

## Methods: participants, interventions and outcomes

### Study setting {9}

Recruitment of people with ABI will take place between November 2019 and July 2020 in three hospitals in the Netherlands: Deventer hospital (Deventer), St. Antonius hospital (Nieuwegein and Woerden) and Flevo hospital (Almere). Hospital staff will recruit people with ABI without further involvement in the study procedures, assessments will take place through home visits, via telephone or by sending questionnaires via mail.

### Eligibility criteria {10}

#### People with ABI

People with ABI are eligible for the trial if they comply with all of the following criteria at hospital discharge:

- Acquired brain injury objectified by medical specialist (meningitis, encephalitis, hydrocephalus, subarachnoid hemorrhage, intracerebral or intracranial hemorrhage, ischemic stroke, transient ischemic attack, concussion, contusion, other head trauma).
- Aged 18 years or older.
- Living in the community prior to the injury.
- Discharged home or to a rehabilitation centre after hospital visit/admission.
- Sufficient command of the Dutch language to understand study procedures.
- Access to a computer and the internet (to use the monitoring tool, see ‘Intervention description {11a}’).
- Willing and able to give informed consent.

Exclusion criteria for people with ABI are:

- A neurodegenerative disorder such as Parkinson’s disease or dementia (because of the progressive course of the disease).
- A diagnosis related to neuro-oncology (since an intensive care trajectory is already in place for this group).
- Discharge to a nursing home.

#### Family members

Family members are eligible when they comply with all of the following below. Off note, we speak of family members since usually the partner, a child or a parent is most likely to be the primary caregiver in case the person with ABI needs support, but friends or neighbours can also participate in this role if they are the ones most close to the person with ABI.

- The person with ABI is eligible and willing to participate (i.e. family members can only participate if their relative with ABI is participating).
- They are (or would be if necessary) the primary informal caregiver; i.e. the person most close the person with ABI.
- Aged 18 years or older.
- Sufficient command of the Dutch language to understand study procedures.
- Access to a computer and the internet (to use the monitoring tool and questionnaires).
- Willing and able to give informed consent.

#### Case managers

Health care professionals are eligible for the role of case manager if they have professional experience in caring for people with ABI at bachelors’ level or higher (e.g. social workers, nurses, speech and language therapists and occupational therapists). They need to be available for at least 4–8 h per week for the duration of the project, willing to participate in the case manager training at the beginning of the project and in the monthly supervision meetings, and willing to register and document their case manager activities and experiences for research purposes. A formal application procedure will be followed; candidates are hired based on their resume, motivation and job interview by the project leaders.

### Who will take informed consent? {26a}

People with ABI and family members who are willing to participate will be visited at home by a trained research assistant, who will obtain informed consent prior to baseline assessment and after going over the study procedures. In case the home visit cannot take place, study procedures will be explained over the phone and informed consent is obtained by mail.

### Additional consent provisions for collection and use of participant data and biological specimens {26b}

On the consent form, participants will be asked if they agree to storage and use of their personal information for future research on brain injury or case management and if they agree to be approached for participation in future studies. By signing the consent form, participants give permission to the use of their data should they choose to withdraw from the study, for the research team to request injury-related information from their medical files and to share data with the regulatory authorities and the clinical research monitor of Maastricht University, where relevant. This trial does not involve collecting biological specimens for storage.

## Interventions

### Explanation for the choice of comparators {6b}

Case management is compared to the usual care as this is the current alternative.

### Intervention description {11a}

#### Case management

##### The framework

The framework for case management for ABI was developed based on a combination of the taxonomy for case management [24] and descriptions of case management for dementia in the Netherlands (e.g. [33–46]), because this form of case management is reasonably well integrated in the Dutch health care system. Such services can serve as a base for care innovations for people with ABI because of the shared focus on supporting people to adapt to the consequences of a disorder or disease in daily life [47].

Case management aims to support people with ABI and family member’s’ self-management of the consequences of ABI and psychosocial well-being, to prevent (escalation of) problems and to facilitate timely access to appropriate services. We propose the following case management elements:

- Monitoring: tracking functioning and well-being of people with ABI and family members. In the present study, a digital monitoring system is used for this purpose (described below).
- Identification: identification of questions, problems and needs (based on monitoring) that hinder functioning and well-being at the time they emerge.
- Assessment: assessing the nature and severity of the presented problem, burden on and capabilities of the person with ABI and the family member, the role of their social network, making implicit or unmentioned questions and problems explicit, drawing conclusions about the core problem in the individual context.
- Information (psycho-education): providing information and education on the (impact of) ABI to assist understanding, information or education related to the question or problem (with a focus on capabilities to self-manage the problems), informing on available care and support services.
- Provision of support: guiding decision-making with regard to managing the problem, providing practical or psychosocial support for relatively mild problems (focused on maintaining or improving self-management).
- Referral: referring to more specialized care or support for relatively complex problems and guiding decision-making with regard to what available services to use.
- Coordination: supporting access to services, facilitating collaboration between different service providers and bringing about appropriate care when this is not available through the regular services.

Case management is person-centred and supposed to follow the ‘stepped care’ and ‘matched care’ principles, starting with the least complex form of care and support that meets the demand for help (stepped care), with the form and intensity individually built around the needs and capabilities of the person with ABI and/or the family member (matched care). Case manager activities may therefore vary from offering a listening ear or providing information and advice, to intensively coordinating longer-lasting specialist care. Case manager involvement also may vary in intensity over time, ranging from only monitoring to more intensive involvement.

Case management activities are community-based; they will take place at peoples’ home or other relevant places such as at work, or over the phone, via video calls or via email. While following the stepped care principle, case managers are flexible in the actions (interventions) they choose based on their own professional expertise, the links they make with available services (e.g. they are independent) and the way they create support when available services do not match people’s needs. Part of this study is mapping actual case manager activities onto the proposed elements and exploring whether this concept should be adjusted based on case manager and participant experiences, in order to move towards a more detailed description of what these elements entail in practice.

##### Monitoring tool (the ReMinder)

Participants receiving case management will be entered into a digital monitoring tool, called the ReMinder, developed by authors KHMJ and MD. This tool was originally developed to empower people who leave the hospital after a head trauma or stroke and their family to find information and get easy access to care in case of the development of problems caused by the injury on the long term. In the current research project, ReMinder is incorporated within OZO Verbindzorg, an online communication system that links different service providers through an online platform. The participant decides who gets access to the information in their OZO Verbindzorg account. For this project, this concerns sharing responses to the ReMinder questions (see below) with their case manager. Participants are in control of their account and may add other professionals involved in their care. They are in control of what information is shared with whom. All procedures are conducted according to the prevalent laws for personal data and privacy.

People with ABI and family members, each having their own account, will automatically receive an email every 3 months, which allows them to enter the monitoring system and to answer two questions: (1) Do you experience problems as a consequence of brain injury? (2) Are you (Is your loved one) able to do all the things you were (he/she was) doing prior to the brain injury? Both questions can be answered with yes or no. If the participant responds ‘yes’ to question 1 and/or ‘no’ to question 2, they are directed to a 32-item questionnaire asking about functioning in the areas of health, daily life, activities, social contacts and consequences of ABI. This questionnaire was composed by the developer of the ReMinder (authors KHMJ and MD), inspired by the Checklist for Cognitive and Emotional Consequence of Stroke (CLCE-24 [48]), the Utrecht Scale for Evaluation of Rehabilitation-Participation (USER-P [49];), Outcome Questionnaire (OQ-45 [50];) and the life domains of the ICF: International Classification of Functioning, Disability and Health (ICF-model). Participants fill out the questionnaires as self-report. Family members fill out the questionnaire reporting about the person with ABI and five extra questions about his or her own well-being. In order to facilitate easy access to the case manager, at completion of the 32-item questionnaire participants will be asked if they would like to have contact with the case manager (yes/no). In addition, people with ABI and family members also have the opportunity to ask a question to the case manager directly at any time within the online environment.

The responses are visible to the case manager to keep track of functioning and well-being of the person with ABI. The case manager will contact those participants who explicitly indicate that they would like to get in touch. For participants who do not initiate contact with their case manager themselves, the case manager determines whether to contact the participant after the second time participants have filled out the ReMinder (i.e. 3 months after the first ReMinder questionnaire). The decision to get in touch with the participant will generally be based on whether responses indicate that certain problems have not been resolved (consistently low scores in one or more areas), a deterioration in scores, or profound discrepancies in scores between the person with ABI and their family member.

##### The case manager

The case manager is a fixed contact person; building a relationship is desirable because of the complexity of learning to live with ABI within the individual context, and to prevent the person with ABI and their family from having to tell their story over and over again. A back-up case manager will be assigned as well, who will be involved when the primary case manager is unavailable and who can serve as a sparring partner for the primary case manager.

The case managers form three teams, one in each region. Teams are composed of professionals from different disciplines and with varying backgrounds; some have been working in clinical/rehabilitation settings, others in community outreach, and their experience with support and treatment approaches may be focused on cure or on care. Two of the teams include a peer support worker.

Case managers will participate in a 4-day training prior to the start of the study. The training has a coaching character, promoting team members to draw upon each other’s knowledge and experience. During the training, case managers learn to see beyond one’s own specific professional discipline, get to know the professional background of the other team members and are coached in learning from each other to best support people with ABI and their family. Regular supervision meetings are organized with case managers within regions at least every 2 months, and between regions twice a year.

#### Care as usual

The usual care differs depending on the regional structures and collaborations. In all regions, limited structured care is available for people who suffered a stroke, mostly for secondary prevention purposes, with a limited duration of 1 year. No structural care is provided for other types of ABI. People with ABI can make use of different forms of care that may or may not involve professionals with expertise on ABI, such as physiotherapy, occupational therapy or social work, but patients usually need to take initiative to find and access these services either themselves or through their general practitioner.

### Criteria for discontinuing or modifying allocated interventions {11b}

By design, case management is a modifiable form of care, as it is about organizing interventions based on the individuals’ needs, strengths, weaknesses and living situation. As described before, this study is of a pragmatic nature, granting case managers the flexibility to act as they see fit to support participants, while providing the least amount of support necessary, to stimulate self-management.

Complete discontinuation of case management will occur on participants’ request. People with brain injury can continue if their family member wishes to stop. Off note, a form of ‘active discontinuation’ occurs when participants do not need any support at a given moment: case managers will then monitor the participants’ well-being via the ReMinder and will be available when questions or problems do occur, but do not reach out otherwise. Following the intention-to-treat principle, participants who choose to withdraw from case management (i.e. the intervention) will still be asked to participated in the study assessments. Outcomes are no longer collected when participants choose to withdraw from the study (i.e. the assessments).

### Strategies to improve adherence to interventions {11c}

The ReMinder serves as the basis for case managers to monitor if support is needed; participants will receive reminder emails twice a week until they open the ReMinder questionnaire. When two consecutive ReMinder questionnaires (i.e. 3 months apart) are not filled out, participants will be asked by email whether they have trouble getting access to the system, with filling out the questions or whether there is another reason for not using the ReMinder. If they do not respond to any of these emails, their case manager will reach out to explore the reason for this and to determine if further case manager activities are required. Other elements of case management are not subject to adherence as such.

### Relevant concomitant care permitted or prohibited during the trial {11d}

All participants are allowed to receive any form of care that they need. Service use will be measured with a questionnaire. Participants are asked not to participate in any other studies concerning psychosocial or pharmacological care for the duration of this trial.

### Provisions for post-trial care {30}

Depending on the results of the study and available funding, case management will be continued or gradually scaled down. In case of continuation, participants in the control condition will have the opportunity to receive case management after the study period.

### Outcomes {12}

#### Effectiveness

Outcomes measures were chosen according to the aim of case management, which involves the concepts of psychosocial well-being, self-efficacy and (unmet) needs. Assessment will take place at baseline, after 6, 12, 18 and 24 months. The total score of the Hospital Anxiety and Depression Scale (HADS [51];) will serve as the primary outcome measure, other measures (see below) are secondary outcomes.

##### Outcomes for people with ABI

Psychosocial well-being will be assessed using the HADS, the Utrecht Scale for Evaluation of Rehabilitation-Participation (USER-P [49]) restriction subscale and the Life Satisfaction Questionnaire (LiSat [52]). The HADS consists of 14 items scored on a 4-point scale ranging from 0 to 3 with varying anchors. Total scores can be calculated for the full scale (primary outcome). The two subscales (anxiety and depression) will also be computed and analysed as secondary outcomes; subscale scores of > 7 suggest the presence of an anxiety disorder or depression. The psychometric quality of the scale is sufficient [53]. The USER-P restriction subscale consists of 9 items asking about restrictions in vocational, leisure and social activities as a consequence of ABI. Items are rated on a scale from 0 (not possible) to 3 (without difficulty) and a ‘not applicable’ option. The total score ranges from 0 to 100 based on the number of applicable items; higher scores indicate less restrictions in participation. The scale has shown sufficient reliability and validity [49]. The LiSat assesses various aspects of life satisfaction including life as a whole, self-care management, contacts with friends, vocational, family life, partner relationships, financial situation, leisure situations and sex life. The nine items are scored on a ﻿6-point scale ranging from ‘very dissatisfied’ to ‘very satisfied’. The scale has satisfactory reliability and validity [54, 55].

The concept of self-efficacy, one’s confidence in the ability to deal with (health) problems, will be measured as a proxy for self-management, since self-efficacy is a prerequisite for behavioural change. Self-efficacy will be measured with the Patient Activation Measure (PAM [56];), which is a 13-item instrument assessing self-reported knowledge, skills and confidence for self-management of one’s health or chronic condition. Items are scored on a scale of 0 (disagree strongly) to 4 (agree strongly) and a not applicable option. An algorithm is available to transform the scores on the PAM to different levels of self-management, from ‘disengaged and overwhelmed’ to being their own health advocate. The PAM requires a license and sharing of the de-identified data with Insignia Health. The Dutch version of the PAM has shown moderate test-retest ability (*r* = 0.47) [57].

Care needs will be assessed with the Longer-term Unmet Needs after Stroke questionnaire (LUNS [58];). The LUNS consists of 22 items scored with ‘yes’ or ‘no’ and one open ended question on the physical, social, and emotional consequences of stroke. To make the LUNS applicable for people with other types of brain injury, we replaced the word ‘stroke’ by ‘brain injury’ in two items. A validation study of the Dutch version of the LUNS concluded that the scale is reliable and valid [59]. It should be noted that some items merely express worries or a problem rather than needs (e.g., ‘I am worried that I might fall [again] and this is stopping me from doing my usual things’). Nevertheless, we consider the scale to be the most comprehensive scale to assess care needs in the ABI population available in Dutch.

##### Family member outcomes

Psychosocial well-being, self-efficacy and (unmet) needs will also be measured in family members. In addition to the HADS and LiSAT, of which a description is provided above, caregiver burden will be assessed within the concept of psychosocial well-being. The Caregiver Strain Index (CSI [60];) will be used, which consists of 13 items that can be responded to with ‘yes’ or ‘no’ and total scores ranging from 0 to 13; higher scores reflecting higher caregiver burden and substantial burden is indicated by a score of 7 or higher. For people who suffered from stroke, the CSI is the most commonly used scale and recommended in the Dutch stroke care guidelines [61]. The scale has shown sufficient validity and reliability [62].

*S*elf-efficacy will be assessed using the Carer Self-Efficacy Scale (CSES) [63]. The CSES measures self-efficacy with regard to care management and service use, each in 5 items on a 10-point scale from ‘not at all certain’ to ‘very certain’; higher scores on the CSES indicate higher levels of self-efficacy. Reliability and validity of the scale are sufficient [64].

Family members’ needs will be assessed with the Family Needs Questionnaire (FNQ [65]). The scale includes 40 items assessing needs that may arise during acute rehabilitation, soon after discharge and in the long-term after ABI. Subscales include health information, emotional support, instrumental support, professional support, community support network and involvement with care. Family members are asked to indicate the importance of each perceived need and then rate the degree to which the need has been met. The Dutch translation has shown sufficient reliability [66]. Information on the validity of the translation is not yet available, but the English version has shown to be valid [67].

#### Cost-effectiveness and cost-utility

Cost-effectiveness and cost-utility will be determined using the EuroQol (EQ-5D-3L) and a service use questionnaire, included in the assessments on baseline and after 6, 12, 18 and 24 months. The EQ-5D-3L [68] consists of five questions measuring health status. The dimensions covered are mobility, self-care, daily activities, pain or discomfort, and anxiety or depression. These domains are rated as ‘no problem’, ‘moderate problem’ or ‘unable to do’. The EQ-5D-3L has shown good measurement properties [69]. Service use will be measured with a self-report cost questionnaire, which was constructed to collect cost data from a societal perspective. It is based on the steps described by Thorn and colleagues [70] and on the questionnaire used by Rauwenhoff and colleagues [71].

#### Feasibility

The assessment of feasibility is of exploratory nature and will be assessed using the process evaluation framework of Saunders, Evans and Joshi [72]. This involves mapping fidelity (quality), dose delivered (completeness), dose received (exposure), reach (participation rate), recruitment (procedures, maintenance of participant involvement) and context (aspects of the physical, social, and political environment). Data will be collected continuously in the form of registrations by case managers, and focus groups will be held at study end (18–24 months after baseline). Data will primarily be used in a summative and descriptive manner. Quantitative indicators to determine feasibility are:

- At least 67% of the participants fills out the ReMinder each wave (every 3 months).
- Case managers respond to at least 90% of the times patients the request for contact and contact patients 90% of the times this is indicated by the responses in the ReMinder.
- Satisfaction with case management (beyond monitoring) is rated with a 7 or higher on a scale of 1–10.
- At least 70% making use of case management (beyond monitoring) would recommend case management to others.

Qualitative indicators are:

- Participants and case managers reporting on case management in terms of it being acceptable, feasible and useful.
- Participants reporting on case manager activities to match their needs (matched care).
- Participants and case managers reporting on increasing support when necessary and taking steps back when possible (stepped care)
- Participants reporting on case managers’ expertise and skills, case managers reporting on feeling well-equipped to appropriately support participants’ needs.

#### Other study parameters

The following demographic and injury-related characteristics will be collected at baseline: date of birth, gender, education, date of most recent ABI, type of most recent ABI, date and type of previous ABI(s), hospital admission (yes/no), length of hospital stay of most recent ABI, referral destination at hospital discharge (home or rehabilitation centre).

### Participant timeline {13}

The schedule of enrolment, interventions and assessments can be found in Table 1. All participants will receive the study questionnaires every 6 months until December 2021. Depending on the time of inclusion (up to June 2020), people with ABI will be followed up for 18 to 24 months. A subsample of people with ABI and family members will be approached for additional participation in focus group interviews, taking place at the end of the study (between October and December 2021).The evaluation form will be sent after 1 year and at the final measurement, which can be 18 or 24 months after baseline assessment depending of the time of enrolment.

**Table 1:**
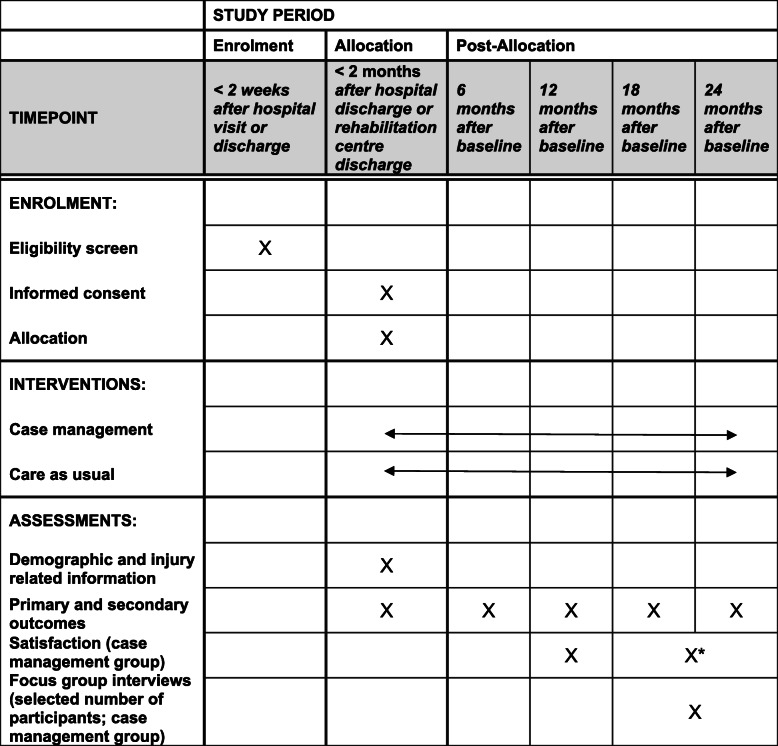
Schedule of enrolment, interventions and assessments

*The evaluation form will be sent after 1 year and at the final measurement, which can be 18 or 24 months after baseline assessment depending of the time of enrolment

### Sample size {14}

Power calculation was based on the primary outcome measure Hospital Anxiety and Depression Scale (HADS). A study evaluating a monitoring/psycho-educational intervention in patients with possible ABI due to cardiac arrest showed to be effective in improving both anxiety and depressive symptoms with a group difference on the HADS of 3.25 points, corresponding to Cohen’s *d* effect size of 0.36 [73]. With an alpha of .05 and power value of 80%, a sample size of 194 is required to detect such between-group effect in the post-intervention measurement. This number can be adjusted for the correlation between baseline and follow-up data, since baseline measures will be entered in the model as an independent variable, by multiplying the sample size by 1 − *R*^2^ (R is the population correlation between the dependent variable (post-intervention) and the pre-intervention measurement) [74]. *R* is estimated to be at least 0.5, making the sample size 194 × (1 − 0.5^2^) = 146. Taking a drop-out rate of 30% into account, at least 209 people with ABI should be recruited. For each participating person with ABI, the family member who is or would be acting as the informal caregiver will be asked to be enrolled in the study as well. The number of participating family members will not exceed the maximum of 209.

### Recruitment {15}

At each of the three recruiting hospitals, trained hospital staff will select eligible people with ABI from the electronic patient files. The hospital staff explains the aim of the study and the study procedures to the patient and ask whether they have a family member who might be interested in participating as well. When the person with ABI or person with ABI-family-member couple is interested in participation, the hospital staff will send them the study information and notifies the researcher. The researcher will call after a week to clarify any questions patients or family members may have. If they are interested in participating, an appointment will be scheduled with the research assistant to sign informed consent, complete the baseline assessment and perform the randomization. In case the person with ABI is referred to inpatient rehabilitation, the appointment will take place as soon as possible after discharge.

## Assignment of interventions: allocation

### Sequence generation {16a}

Participants (people with ABI or people with ABI-family-member couples) will be randomly allocated with a 1:1 ratio to either the case management group or the care as usual group, using a computerized random schedule, in blocks of six. The randomization block size will not be disclosed to the research assistant who enrols and assess participants, to ensure concealment.

### Concealment mechanism {16b}

The research assistant will be provided with sequentially numbered, opaque sealed envelopes containing randomization information.

### Implementation {16c}

The allocation sequence will be generated by a person who is not involved in the study assessments, using a computerized random number generator (www.random.org/lists/). This person will prepare sequentially numbered opaque envelopes containing the information about the group assignment of the participant and seal and the envelopes. The research assistant, who is blind for the allocation sequence and block size, will enrol participants, open the envelope after baseline assessment is completed and provide participants with the information about treatment allocation.

## Assignment of interventions: blinding

### Who will be blinded {17a}

At baseline assessment, group allocation will be unknown to both the research assistant and the participants; treatment allocation takes place after completion of the baseline assessment by a research assistant. If people with ABI wish to be visited at home for the follow-up assessments rather than receiving the questionnaires via mail, they will be visited by a member of the research team who is blind to their treatment allocation. Blinding is not possible for the assessment of feasibility.

### Procedure for unblinding if needed {17b}

There are no foreseen circumstances under which unblinding is necessary.

## Data collection and management

### Plans for assessment and collection of outcomes {18a}

#### Feasibility

Several methods will be used to obtain information on the process evaluation outcomes: (1) registration forms for case manager activities, (2) written notes of supervision meetings, (3) responses to the questionnaires and participants’ communication with case managers derived from the ReMinder and the OZO system, (4) evaluation forms after 12 months and at study end for participants (both people with ABI and family members) receiving case management and (5) focus group interviews at study end.

##### Focus groups

Focus groups will be held to obtain in-depth information on the experiences with receiving case management (for participants) and with delivering case management (for case managers). Focus groups are group interviews (approximately 6 participants each) with a particular subject (or focus), and they make use of social interactions between participants. Focus groups are an ideal method to reveal various perspectives on a topic and to uncover new insights and unanticipated issues [75]. During the focus groups, a moderator (researcher) will use a discussion guide that will include questions on the topics based on fidelity, dose delivered, dose received, reach, recruitment and context [72]. The interviews will be audio recorded as well as video recorded, to ensure identification of potentially relevant non-verbal information or cues presented by the participants. A second researcher will take additional notes during the focus group interviews.

People with ABI and family members of the three different regions will be selected to form a sample including a wide range of injury-related characteristics and needs (purposively selected) to participate in focus group interviews at the end of the study period. In each region, one people with ABI focus group and one family member focus group will be planned (six in total). This should be sufficient to achieve saturation, i.e. when no new issues emerge from the last focus group, as research suggests that saturation is usually achieved after the fourth group discussion [75]. Saturation will be checked after these groups and if necessary, additional focus groups will be planned. Because the case managers form a limited group within the scope of the project (25–30 case managers), we aim to include all case managers in focus groups, divided in 3–4 groups.

#### Effectiveness and cost-effectiveness

The baseline assessment will be completed no more than 2 months after the visit to or discharge from the hospital or, in case of inpatient rehabilitation following hospital discharge, as soon as possible after they leave the rehabilitation centre. Ideally, assessments take place at the participants’ home, but can take place via telephone or questionnaires may be sent and returned by mail in case home visits are not possible (on time). A trained research assistant will explain the study procedures once more and collect the consent form. During home visits, participants fill out the questionnaires on their own and have the opportunity to ask questions. The research assistant will provide support when necessary, for example by reading the questions and possible answers out loud in case of reading difficulties (a common problem after stroke). Information on gender, educational level and living situation will be collected at baseline; date of birth, type and date of most recent and previous brain injuries and discharge destination will be drawn from hospital records by hospital staff. Follow-up measurements will be mailed to the participants, unless people prefer to be assisted, in which case a research assistant (blind to treatment allocation) will visit them at home.

### Plans to promote participant retention and complete follow-up {18b}

Participants will receive a five-euro gift card for each measurement (maximum of 25 euros for the complete study). For participating in the focus groups, they will receive an additional fee of ten euros (gift card).

### Data management {19}

Data will be collected on paper and then entered electronically twice by members of the research team; any discrepancies between the two entries will be resolved by the person responsible for the second entry, or by discussion with a third researcher if necessary. There are restrictions in place for entering questionnaire data based on the range of answers. Manual range checks will be performed for demographic information. An audit trail will provide with information on all activities in the electronic database. Access to electronic data is controlled by a password system, and access to original data will be restricted by storing the data in a locked cabinet (see ‘Confidentiality {27}’).

### Confidentiality {27}

Data will be handled confidentially and reporting will be coded. All participants will receive a unique identifier that cannot be used to link the data to an individual subject (i.e. CM001). Collected data and personal information will be stored separately in locked cabinets. The involved researchers from Maastricht University will safeguard the key to the code. Only the national supervisory authorities such as the Inspection for Healthcare and Youth (in Dutch: Inspectie voor Gezondheidszorg en Jeugd) will have access to the data upon request. The handling of personal data will comply with the EU General Data Protection Regulation (GDPR) (in Dutch: AVG), the Dutch Act on Implementation of the General Data Protection Regulation, and the Research Data Management Code of Conduct of Maastricht University. The data will be stored for 15 years after the end of the study.

### Plans for collection, laboratory evaluation and storage of biological specimens for genetic or molecular analysis in this trial/future use {33}

There will be no collection of biological specimens.

## Statistical methods

### Statistical methods for primary and secondary outcomes {20a}

#### Effectiveness

Multilevel modelling will be used to assess the improvement of case management participants over time compared to care as usual. Primary analysis involves entering time, group and their interaction (exposure) as fixed terms. Separate analyses will be performed to assess 24-month outcomes for the subgroup of participants for whom this data is available. Sensitivity analysis will be performed by extending the resulting models with covariates, controlling for the effects of age, gender and level of education. Covariates will be kept in the model as long as they contribute significantly to the model. Significance of the fixed regression effects will be tested using the appropriate *t*-test (*α* = .05).

#### Cost-effectiveness and cost-utility

The trial-based economic evaluation will involve a combination of a cost-effectiveness analysis (CEA) and a cost-utility analysis (CUA). Effects will be presented as clinical outcomes (i.e. self-efficacy and psychosocial well-being). In these CEAs, the incremental cost-effectiveness ratio (ICER) will be expressed as the incremental costs per point improvement on the primary outcome measure. The primary outcomes measure for the CUA will be quality-adjusted life years (QALYs), based on the EuroQol (EQ-5D-3L). The EQ-5D-3L can distinguish between different health states. For each of the different states, a weight is contributed based on the valuation given by the general population (Euroqol group). These range from 0 (representing death) to 1 (representing full health). Cost-effectiveness evaluations make use of these utilities. In the CUA, the ICER will be expressed as the incremental costs per QALY. This economic evaluation will be performed from a societal perspective, which implies that all relevant costs and outcomes will be considered. The time horizon will be the same period as the follow-up period of the trial.

Total costs will be estimated using a bottom-up (or micro-costing) approach, where information on each element of service used is multiplied by an appropriate unit cost and summed to provide an overall total cost. The economic evaluation will assess not only the intervention costs, but also healthcare costs, patient and family costs, and costs outside the health care sector. For this study, we have developed a cost questionnaire, based on existing questionnaires which will identify all relevant costs aspects.

Despite the usual skewness in the distribution of costs, the arithmetic means will be generally considered the most appropriate measures to describe cost data. In case of skewness of the cost data, non-parametric bootstrapping will be used to test for statistical differences in costs between the case management and care as usual group. The bootstrap replications will be used to calculate 95% confidence intervals (CI) around the costs (95% CI). If cost data are distributed normally, *t*-tests will be used. The robustness of the ICER will be checked by non-parametric bootstrapping (1000 times). Bootstrap simulations will also be conducted in order to quantify the uncertainty around the ICER, yielding information about the joint distribution of cost and effect differences. The bootstrapped cost-effectiveness ratios will be subsequently plotted in a cost-effectiveness plane, in which the vertical line reflects the difference in costs and the horizontal line reflects the difference in effectiveness. The choice of treatment depends on the maximum amount of money that society is prepared to pay for a gain in effectiveness, which is called the ceiling ratio. Therefore, the bootstrapped ICERs will also be depicted in a cost-effectiveness acceptability curve showing the probability that case management is cost-effective using a range of ceiling ratios.

#### Feasibility

Registration forms, evaluation forms and written notes of supervision meetings will be analysed descriptively, and focus groups will be analysed qualitatively. Audio and videotapes of all focus group interviews will be transcribed verbatim. Analyst triangulation will be applied [76]; the transcripts and observations combined with additional notes that will be taken during the focus group interviews will be analysed independently by two researchers using the qualitative analysis software ATLAS.ti (version 7.0). An inductive content analysis approach will be adopted [77], in which common themes and categories emerge using inductive reasoning and constant comparison. The texts will be thoroughly read and open codes will be applied to describe all aspects of the content [78]. Codes referring to the same phenomenon will be grouped into categories and these categories will be grouped into higher-order themes. Categories and themes will be combined into general statements to describe the phenomenon [77]. Discrepancies in coding and interpretation will be discussed in a meeting together with a third researcher to reach consensus regarding the categories and themes. The video recordings will be compared with the written transcripts to be able to identify potentially relevant additional non-verbal information or cues presented by the participants. Quotations will be selected based on representativeness of the emerged themes by the coordinating researcher and verified by the other two researchers.

#### Other study parameters

Demographic and injury-related characteristics will be analysed descriptively.

### Interim analyses {21b}

No interim analyses are planned because there are no anticipated risks to participation in this study.

### Methods for additional analyses (e.g. subgroup analyses) {20b}

Subgroups will be formed based on type of injury (stroke vs. other types) and severity, for which length of hospital stay will be used as a proxy (based on the distribution of the sample). Even though the subgroups are likely to be too small to draw firm conclusions, we will analyse this exploratively because the usual care for stroke is already better organized than for other types of brain injury (possibly lowering the additional gains of case management over care as usual for stroke compared to other types of brain injury) and because those with moderate to severe ABI may have more to gain from case management than those with mild ABI.

### Methods in analysis to handle protocol non-adherence and any statistical methods to handle missing data {20c}

Data will be analysed by the intention-to-treat approach. Sensitivity analysis will be performed with regard to missing data; we will evaluate which of the measured variables are associated with missing outcomes and will include these in the model. Reasons for drop-out or missing assessments will be documented if participants are willing to share this.

### Plans to give access to the full protocol, participant level-data and statistical code {31c}

The full protocol, anonymized data set and statistical code will be available on request after the results of the study have been published.

## Oversight and monitoring

### Composition of the coordinating centre and trial steering committee {5d}

#### Principal (CMvH), coordinating investigators (APMS; CR interim)

- Design of the study;
- Preparation of protocol and revisions;
- Ethics committee application;
- Study planning;
- Recruiting, training and supervising research assistants;
- Responsible for trial master file;
- Provide annual reports to ethics committee;
- Data verification;
- Publication of study reports;

#### Project team

Principal (CvH) and coordinating (APMS; CR interim) investigators, overall project leader (JZ), intervention project leader (NJ), ReMinder monitoring tool project leader (KJ, MD)

- Agreement of final protocol;
- Recruiting hospital staff and assistance with recruiting procedures;
- Implementing ReMinder;
- Entering participants in case management group into monitoring tool;
- Recruitment, training and supervising case managers;
- Collecting registrations of case management activities;
- Organizing project team meetings.

### Composition of the data monitoring committee, its role and reporting structure {21a}

Because of the low burden and minimal risks, no data monitoring committee was appointed.

### Adverse event reporting and harms {22}

We will only report those adverse events that are directly related to our study, defined as experiencing negative consequences of case management in terms of psychosocial well-being or self-management that are reported spontaneously by the subject. Due to the non-invasive nature of this study, no experiment-related (serious) adverse events are expected.

### Frequency and plans for auditing trial conduct {23}

As this study falls under the scope of the Dutch Medical Research Involving Human Subjects Act (Dutch: wet medisch-wetenschappelijk onderzoek met mensen, WMO), the Clinical Trial Centre Maastricht (Maastricht University) appointed an independent clinical research monitor to the study. This person monitors whether the study is conducted according to the ICH-GCP guidelines and legislation and regulations. For our study, four visits divided over the study period are planned.

Activities of the clinical trial monitor are:

- Giving advice regarding laws and regulations;
- General control of data collection;
- Verification of source documents and CRFs;
- Controlling the compliance of laws and regulations;
- Complying all protocols;
- Checking of informed consents;
- Controlling the Trial Master File;
- Verifying the reports on adverse events and complications.

### Plans for communicating important protocol amendments to relevant parties (e.g. trial participants, ethical committees) {25}

All changes (substantial and non-substantial) made to the study protocol after the favourable opinion was given by the accredited medical ethics committee (Medical Ethics Committee of Maastricht University Medical Center+) will be notified to the medical ethics committee, documented in the trial registration and communicated in the publication of the results of this study.

### Dissemination plans {31a}

The results, whether positive or negative, will be disclosed unreservedly and submitted for publication to peer-reviewed scientific journals and presented on national and international conferences and meetings for healthcare professionals and people with ABI.

## Discussion

It is essential that people with ABI are supported in learning how to live with ABI, within the individual context in terms of home, education, work, relationships, stage of life and personal goals [14]. Creating a continuous chain of support from hospital discharge onwards is high on the agenda of guidelines and action plans for different types of ABI [12–15]. Continuous and long-term support is currently lacking, as is evident from numerous studies reporting this an important unmet need for people with ABI and caregivers [9–11] and the lack of randomized controlled trials on longer-term care. We respond to this issue by developing and evaluating case management for people with ABI and their family members. We will evaluate whether case management for ABI is feasible, effective and cost-effective compared to care as usual with a randomized controlled trial.

Strengths of the study are the pragmatic nature of the study and the long-term follow-up. The follow-up with a maximum of 24 months approximates the time it usually takes for people to regain a balance in their lives [10]. Furthermore, the pragmatic nature allows us to evaluate case management the way it could be implemented in regular practice. The use of the monitoring tool (the ReMinder) is another strength, as it takes a minimum amount of time and effort to keep track of a large group of people for long periods of time. Finally, the combination of qualitative and quantitative evaluation methods should provide us with rich data on feasibility, effectiveness and cost-effectiveness.

A possible limitation of our study is that for a complete picture of cost-effectiveness and cost-utility of case management, the limits placed on the time frame of our study (18–24 months) will be sufficient to capture a long-term reduction in costs. That is, we expect costs to rise in the first year(s) as case management aims to support in getting timely access to services, while the expected longer-term reduction in costly intensive support may extend our study period. Another possible limitation is that we did not define inclusion criteria with regard to severity of ABI, which means that we will include people with mild ABI who may recover well without support; if this group turns out to be large, they may end up masking effects for the more severe group who benefits from case management. Nevertheless, we deliberately chose to include this group, because care continuity for people experiencing problems after mild ABI is currently missing. Case management could fill this gap; by monitoring people with mild ABI, those with suboptimal recovery can be identified and supported, while those who do fully recover require little case manager time, effort and costs.

By evaluating case management for ABI, this study aims to move forward in bridging the gap between the available care and the needs of people with ABI and their family members. If our study shows promise for case management to be (cost)-effective and feasible, it could be a valuable form of regular care to support people with ABI and their family members in finding a new balance in life.

## Trial status

The Medical Ethics Committee of Maastricht University Medical Center+ granted ethics approval of the third version of the protocol on September 17, 2019. The trial was registered at the Netherlands Trial Register (registration number NL8104, https://www.trialregister.nl/trial/8104) on October 22, 2019, after which recruitment started. The first person was enrolled on November 25, 2019. Inclusion is currently ongoing and expected to be completed in September 2020.

## Abbreviations

CEA: Cost-effectiveness analysis
CI: Confidence interval
CSES: Carer Self-Efficacy Scale
CSI: Caregiver Strain Index
CUA: Cost-utility analysis
FNQ: Family Needs Questionnaire
GDPR: General Data Protection Regulation
HADS: Hospital Anxiety and Depression Scale
ICER: Incremental cost-effectiveness ratio
LiSat: Life Satisfaction Questionnaire
LUNS: Longer-term Unmet Needs after Stroke
PAM: Patient Activation Measure
QALY: Quality-adjusted life years
USER-P: Utrecht Scale for Evaluation of Rehabilitation-Participation

## References

[1] Allanson F, Pestell C, Gignac GE, Yeo YX, Weinborn M (2017). Neuropsychological predictors of outcome following traumatic brain injury in adults: a meta-analysis. Neuropsychol Rev.

[2] Jokinen H, Melkas S, Ylikoski R, Pohjasvaara T, Kaste M, Erkinjuntti T (2015). Post-stroke cognitive impairment is common even after successful clinical recovery. Eur J Neurol.

[3] De Wit L, Theuns P, Dejaeger E, Devos S, Gantenbein AR, Kerckhofs E (2017). Long-term impact of stroke on patients’ health-related quality of life. Disabil Rehabil.

[4] Bieńkiewicz MMN, Brandi ML, Hughes C, Voitl A, Hermsdörfer J (2015). The complexity of the relationship between neuropsychological deficits and impairment in everyday tasks after stroke. Brain Behav.

[5] Murray J, Young J, Forster A, Ashworth R (2003). Developing a primary care-based stroke model: the prevalence of longer-term problems experienced by patients and carers. Br J Gen Pract.

[6] Jennekens N, Dierckx de Casterlé B, Dobbels F (2010). A systematic review of care needs of people with traumatic brain injury (TBI) on a cognitive, emotional and behavioural level. J Clin Nurs.

[7] Blake H (2014). Caregiver stress in traumatic brain injury. Int J Ther Rehabil.

[8] Anderson MI, Simpson GK, Morey PJ (2013). The impact of neurobehavioral impairment on family functioning and the psychological well-being of male versus female caregivers of relatives with severe traumatic brain injury: multigroup analysis. J Head Trauma Rehabil.

[9] Stokman M, Verhoeff H, Heineke D (2011). Navigeren naar herstel.

[10] Stiekema APM, Winkens I, Ponds R, De Vugt ME, Van Heugten CM (2020). Finding a new balance in life: a qualitative study on perceived long-term needs of people with acquired brain injury and partners. Brain Inj.

[11] Pindus DM, Mullis R, Lim L, Wellwood I, Rundell AV, Aziz NAA (2018). Stroke survivors’ and informal caregivers’ experiences of primary care and community healthcare services – a systematic review and meta-ethnography. PLoS One.

[12] Ontario Neurotrauma Foundation (2016). Clinical practice guideline for the rehabilitation of adults with moderate to severe TBI [Internet].

[13] Winstein CJ, Stein J, Arena R, Bates B, Cherney LR, Cramer SC (2016). Guidelines for adult stroke rehabilitation and recovery: a guideline for healthcare professionals from the American Heart Association/American Stroke Association. Stroke.

[14] Norrving B, Barrick J, Davalos A, Dichgans M, Cordonnier C, Guekht A (2018). Action plan for stroke in Europe 2018–2030. Eur Stroke J.

[15] Sacco RL, Sandercock P, Endres M, Feigin V, Pandian J, Shinohara Y (2015). Review and prioritization of stroke research recommendations to address the mission of the World Stroke Organization: a call to action from the WSO Research Committee. Int J Stroke.

[16] Vroomen JMN, Bosmans JE, Eekhout I, Joling KJ, Van Mierlo LD, Meiland FJM (2016). The cost-effectiveness of two forms of case management compared to a control group for persons with dementia and their informal caregivers from a societal perspective. PLoS One.

[17] Reilly S, Malouf R, Hoe J, Toot S, Challis D, Orrell M (2015). Case management approaches to home support for people with dementia. Cochrane Database Syst Rev.

[18] Joo JY, Liu MF (2019). Effectiveness of nurse-led case management in cancer care: systematic review. Clin Nurs Res.

[19] Norris SL, Nichols PJ, Caspersen CJ, Glasgow RE, Engelgau MM, Jack L (2002). The effectiveness of disease and case management for people with diabetes: a systematic review. Am J Prev Med.

[20] Joo JY, Huber DL (2012). An integrative review of case management for diabetes. Prof Case Manag.

[21] Boger EJ, Demain S, Latter S (2013). Disability and rehabilitation self-management: a systematic review of outcome measures adopted in self-management interventions for stroke. Disabil Rehabil.

[22] Lukersmith S, Millington M, Salvador-Carulla L (2016). What is case management? A scoping and mapping review. Int J Integr Care.

[23] van Mierlo LD, MacNeil-Vroomen J, Meiland FJM, Joling KJ, Bosmans JE, Dröes RM (2016). Implementatie en (kosten-)effectiviteit van casemanagement voor mensen met dementie en hun mantelzorgers: resultaten van de COMPAS-studie. Tijdschr Gerontol Geriatr.

[24] Lukersmith S, Fernandez A, Millington M, Salvador-Carulla L (2016). The brain injury case management taxonomy (BICM-T); a classification of community-based case management interventions for a common language. Disabil Health J.

[25] Malec JF, Buffington ALH, Moessner AM, Thompson JM (1995). Maximizing vocational outcome after brain injury: integration of medical and vocational hospital-based services. Mayo Clin Proc.

[26] Malec JF, Buffington ALH, Moessner AM, Degiorgio L (2000). A medical/vocational case coordination system for persons with brain injury: an evaluation of employment outcomes. Arch Phys Med Rehabil.

[27] Ashley MJ, Persel CS, Lehr RP, Feldman B, Krych DK (1994). Post-acute rehabilitation outcome: relationship to case-management techniques and strategy. J Insur Med.

[28] Heinemann AW, Corrigan JD, Moore D (2004). Case management for traumatic brain injury survivors with alcohol problems. Rehabil Psychol.

[29] Lannin NA, Laver K, Henry K, Turnbull M, Elder M, Campisi J (2014). Effects of case management after brain injury: a systematic review. NeuroRehabilitation..

[30] Fens M, Van Heugten CM, Beusmans G, Metsemakers J, Kester A, Limburg M (2014). Effect of a stroke-specific follow-up care model on the quality of life of stroke patients and caregivers: a controlled trial. J Rehabil Med.

[31] Boter H (2004). Multicenter randomized controlled trial of an outreach nursing support program for recently discharged stroke patients. Stroke..

[32] Mayo NE, Scott S (2011). Evaluating a complex intervention with a single outcome may not be a good idea: an example from a randomised trial of stroke case management. Age Ageing.

[33] Alzheimer Nederland & Vilans (2013). Zorgstandaard dementie.

[34] Groenewoud H, Egers I, Pool A, de Jange J (2008). Evaluatieonderzoek van de pilot casemanagement dementie in de regio Delft Westland Oostland 2005–2007.

[35] Huijsman R, Jansen G, Bolle F (2017). Expertisegebied dementieverpleegkundige (voorheen casemanager dementie).

[36] Ketelaar N, Jukema J, van Bemmel M, Adriaansen M, Smits C. Casemanagement dementie. Methodisch werken en positionering in de keten. Een werk- methodiek ontwikkeld door drie regionale dementieketens. Zwolle; 2015.

[37] de Lange J (2014). Casemanager dementie: een complexe baan. Tijdschr voor Prakt.

[38] de Lange J, Deusing E, Peeters J, Francke A, Pot AM (2013). De kunst van casemanagement. Tien succesfactoren volgens mantelzorgers.

[39] Peeters JM, Francke AL, Pot AM. Organisatie en invulling van casemanagement dementie in Nederland. Verslaglegging van een landelijke peiling onder regionale projectleiders. Utrecht; 2011.

[40] Peeters J, Werkman W, Francke AL (2012). Dementiemonitor Mantelzorg: problemen, zorgbehoeften, zorggebruik en oordelen van mantelzorgers.

[41] Peeters JM, de Lange J, van Asch I, Spreeuwenberg P, Veerbeek M, Pot AM, Francke AL. Landelijke evaluatie van casemanagement dementie. Utrecht; 2012.

[42] Rijken E, Jansen P, Diermanse I, Ten Hove S (2016). Casemanagement dementie: stand van zaken, knelpunten en oplossingen.

[43] Verkade PJ, van Meijel B (2011). Tien jaar casemanagement bij dementie. Tijdschr voor Verpleegkundigen.

[44] Verberne-Nuijten D, de Lange J (2014). Casemanagement in de dementieketen.

[45] Verkade PJ, Kuipers T, van Wees C, Mieremet W, Lenselink J. Expertisegebied casemanager dementie. Utrecht; 2012.

[46] Winters J (2018). Expertiseprofiel casemanagers dementie sociaal werk zorg.

[47] Stiekema APMM, van Heugten CM, de Vugt ME (2019). Joining forces to improve psychosocial care for people with cognitive deficits across diagnoses: social health as a common framework. Aging Ment Health.

[48] van Heugten C, Rasquin S, Winkens I, Beusmans G, Verhey F (2007). Checklist for cognitive and emotional consequences following stroke (CLCE-24): development, usability and quality of the self-report version. Clin Neurol Neurosurg.

[49] van der Zee CH, Visser-Meily JMA, Lindeman E, Jaap Kappelle L, Post MWM (2013). Participation in the chronic phase of stroke. Top Stroke Rehabil.

[50] Lambert MJ, Burlingame GM, Umphress V, Hansen NB, Vermeersch DA, Clouse GC (1996). The reliability and validity of the outcome questionnaire. Clin Psychol Psychother.

[51] Spinhoven P, Ormel J, Sloekers PPA, Kempen GIJM, Speckens AEM, van Hemert AM (1997). A validation study of the Hospital Anxiety And Depression Scale (HADS) in different groups of Dutch subjects. Psychol Med.

[52] Fugl-Meyer AR, Bränholm I-B, Fugl-Meyer KS (1991). Happiness and domain-specific life satisfaction in adult northern swedes. Clin Rehabil.

[53] Bjelland I, Dahl AA, Haug TT, Neckelmann D (2002). The validity of the hospital anxiety and depression scale. J Psychosom Res.

[54] Post MW, Van Leeuwen CM, Van Koppenhagen CF, De Groot S (2012). Validity of the life satisfaction questions, the life satisfaction questionnaire, and the satisfaction with life scale in persons with spinal cord injury. Arch Phys Med Rehabil.

[55] Boonstra AM, Reneman MF, Stewart RE, Balk GA (2012). Life satisfaction questionnaire (Lisat-9). Int J Rehabil Res.

[56] Hibbard JH, Stockard J, Mahoney ER, Tusler M (2004). Development of the patient activation measure (PAM): conceptualizing and measuring activation in patients and consumers. Health Serv Res.

[57] Rademakers J, Nijman J, Van Der Hoek L, Heijmans M, Rijken M (2012). Measuring patient activation in the Netherlands: translation and validation of the American short form Patient Activation Measure (PAM13). BMC Public Health.

[58] Forster A (2013). Validation of the longer-term unmet needs after stroke (LUNS) monitoring tool: a multicentre study. Clin Rehabil.

[59] Groeneveld IF, Arwert HJ, Goossens PH, Vliet Vlieland TPM (2018). The longer-term unmet needs after stroke questionnaire: cross-cultural adaptation, reliability, and concurrent validity in a Dutch population. J Stroke Cerebrovasc Dis.

[60] Robinson B (1983). Validation of a caregiver strain index. J Gerontol.

[61] Nederlandse Vereniging voor Neurologie (2017). Herseninfarct en hersenbloeding.

[62] Sullivan MT (2002). Caregiver strain index (CSI). J Gerontol Nurs.

[63] Fortinsky RH, Kercher K, Burant CJ (2002). Measurement and correlates of family caregiver self-efficacy for managing dementia. Aging Ment Health.

[64] Lorig K, Holman HR (2003). Self-management education: History, definition, outcomes , and mechanisms. Ann Behav Med.

[65] Kreutzer JS, Wehman P (1990). Community integration following traumatic brain injury.

[66] Dalemans R, Overländer S, Knors A. Family needs questionnaire Vertaling naar het Nederlands, onderzoek naar de begrijpelijkheid: Logop en Foniatr; 2011. p. 82–6.

[67] Kreutzer JS, Serio CD, Bergquist S (1994). Family needs after brain injury: a quantitative analysis. J Head Trauma Rehabil.

[68] Herdman M, Gudex C, Lloyd A, Janssen M, Kind P, Parkin D (2011). Development and preliminary testing of the new five-level version of EQ-5D (EQ-5D-5L). Qual Life Res.

[69] Janssen MF, Pickard AS, Golicki D, Gudex C, Niewada M, Scalone L (2013). Measurement properties of the EQ-5D-5L compared to the EQ-5D-3L across eight patient groups: a multi-country study. Qual Life Res.

[70] Thorn JC, Coast J, Cohen D, Hollingworth W, Knapp M, Noble SM (2013). Resource-use measurement based on patient recall: issues and challenges for economic evaluation. Appl Health Econ Health Policy.

[71] Rauwenhoff J, Peeters F, Bol Y, Van Heugten C (2019). The BrainACT study: acceptance and commitment therapy for depressive and anxiety symptoms following acquired brain injury: study protocol for a randomized controlled trial. Trials..

[72] Saunders RP, Evans MH, Joshi P (2005). Developing a process-evaluation plan for assessing health promotion program implementation: a how-to guide. Health Promot Pract.

[73] Moulaert VRMP, van Heugten CM, Winkens B, Bakx WGM, de Krom MCFTM, Gorgels TPM (2015). Early neurologically-focused follow-up after cardiac arrest improves quality of life at one year: a randomised controlled trial. Int J Cardiol.

[74] Rausch JR, Maxwell SE, Kelley K (2003). Analytic methods for questions pertaining to a randomized pretest, posttest, follow-up design. J Clin Child Adolesc Psychol.

[75] Hennink M (2007). International focus group research. A handbook for the health and social sciences.

[76] Patton M (2002). Qualitative research & evaluation methods.

[77] Elo S, Kyngäs H (2008). The qualitative content analysis process. J Adv Nurs.

[78] Hsieh HF, Shannon SE (2005). Three approaches to qualitative content analysis. Qual Health Res.

